# Impact of the COVID-19 pandemic on aortic valve replacement procedures in Germany

**DOI:** 10.1186/s12872-023-03213-y

**Published:** 2023-04-06

**Authors:** Adrian Heidenreich, Peter Stachon, Vera Oettinger, Ingo Hilgendorf, Timo Heidt, Jonathan Rilinger, Manfred Zehender, Dirk Westermann, Constantin von zur Mühlen, Klaus Kaier

**Affiliations:** 1 Medical Centre, Department of Cardiology and Angiology, Faculty of Medicine, University of Freiburg, University Heart Centre Freiburg – Bad Krozingen, University of Freiburg, Freiburg, Germany; 2 Centre of Big Data Analysis in Cardiology (CeBAC), Department of Cardiology and Angiology, Faculty of Medicine, University of Freiburg, Freiburg, Germany; 3 Institute of Medical Biometry and Medical Informatics, Faculty of Medicine, University Medical Centre Freiburg, University of Freiburg, Freiburg, Germany

**Keywords:** TAVI, Covid-19, sAVR

## Abstract

**Background:**

COVID-19 has caused the deferral of millions of elective procedures, likely resulting in a backlog of cases. We estimate the number of postponed surgical aortic valve replacement (sAVR) and transcatheter aortic valve replacement (TAVR) procedures during the first two waves of the COVID-19 pandemic in Germany.

**Methods:**

Using German national records, all isolated TAVR and sAVR procedures between 2007 and 2020 were identified. Using weekly TAVR and sAVR procedures between 2017 and 2019, we created a forecast for 2020 and compared it with the observed number of procedures in 2020.

**Results:**

In Germany, a total of 225,398 isolated sAVR and 159,638 isolated TAVR procedures were conducted between 2007 and 2020 that were included in our analysis. The reduction in all AVR procedures (sAVR and TAVR) for the entire year 2020 was 19.07% (95%CI: 15.19–22.95%). During the first wave of the pandemic (week 12–21), the mean weekly reduction was 32.06% (23.44–40.68%) and during the second wave of the pandemic (week 41–52), the mean weekly reduction was 25.58% (14.19–36.97%). The number of sAVR procedures decreased more than the number of TAVR procedures (24.63% vs. 16.42% for the entire year 2020).

**Conclusion:**

The first year of the COVID-19 pandemic saw a substantial postponing of AVR procedures in Germany. Postponing was higher for sAVR than for TAVR procedures and less pronounced during the second wave of the COVID-19 pandemic.

**Supplementary Information:**

The online version contains supplementary material available at 10.1186/s12872-023-03213-y.

## Introduction

The German government announced the first quarantine restrictions due to the beginning COVID-19 pandemic on 16 March 2020. In order to conserve hospital resources, especially intensive care capacity, the healthcare system was advised to postpone elective procedures for COVID-19 patients. The shutdown affected routine hospital services for all non-COVID patients, who had non-emergency treatments cancelled or significantly delayed. The impact on the quality of life and clinical outcomes of the non-COVID-19 patients who were affected by the COVID-19 lockdown regulations around the world is unknown [1]. Although restrictions and recommendations were eventually lifted, surveys suggest that even patients with life-threatening conditions may have avoided hospital admission, possibly due to fear of SARS-CoV2 exposure [2, 3].

Aortic stenosis is a common valvular heart disease and, while many patients with aortic stenosis are asymptomatic, the onset of symptoms is associated with rapid deterioration. Thus, timely treatment by either surgical aortic valve replacement (sAVR) or transcatheter aortic valve replacement (TAVR) is crucial [4].

This study analyses the impact of the COVID-19 pandemic on the number of sAVR and TAVR procedures conducted in 2020, using healthcare data to estimate the proportion of postponed aortic valve replacement procedures during the first and second waves of the COVID-19 pandemic. Since there is seasonal variation in the number of procedures performed, we compared the number of weekly treatments of aortic valve stenosis in 2020 with the pre-COVID-19 levels in 2007–2019.

## Methods

### Data and measures

All methods were carried out in accordance with relevant guidelines and regulations in the Declaration of Helsinki. Since 2005, data on all hospitalizations in Germany have been available for scientific use via the Diagnosis Related Groups statistics collected by the Research Data Centre of the Federal Bureau of Statistics (DESTATIS). These hospitalization data, including diagnoses and procedures, are a valuable source of representative nationwide data on the in-hospital treatment of patients [5–9]. This database represents a virtually complete collection of all hospitalizations in German hospitals that are reimbursed according to the Diagnosis Related Groups system. From this database, we extracted data on all isolated sAVR and TAVR procedures conducted between 2007 and 2020 [10, 11]. Isolated procedures were defined using OPS codes with the inclusion of all aortic valve procedures and the exclusion of concomitant procedures at the mitral valve, tricuspid valve procedures, coronary artery bypass graft procedures and Maze procedures [10]. Patients with a baseline diagnosis of pure aortic regurgitation (main or secondary diagnosis other than ICD, Tenth Revision (ICD-10) codes I35.0, I35.2, I06.0, I06.2) and those with concomitant cardiac surgery or percutaneous coronary intervention were not included in this analysis, as described previously [10]. All summary results were anonymized by DESTATIS. In practice, this means that any information allowing the drawing of conclusions about a single patient or a specific hospital was censored by DESTATIS to guarantee data protection.

### Statistical analysis

The expected number of AVR procedures in 2020 was calculated for TAVR and sAVR procedures separately. In detail, these TAVR and sAVR forecasts were calculated by application of linear regression models with the number of weekly AVR procedures as endpoints and the calendar year and the calendar week as continuous and categorical covariates, respectively. These regression models included all procedures from 2007 up to and including 2019 and were then used for prediction of the number of AVR procedures expected in 2020. Finally, the expected number of AVR procedures in 2020 was then compared to the actually observed number of AVR procedures in 2020. See Supplemental Table 1 for details of the two regression models. When visualizing the results, calendar week 53 and 1 are omitted due to problems regarding the overlap between years. All analyses were carried out using Stata 16.0 (StataCorp, College Station, Texas, USA).

## Results

**Fig. 1:**
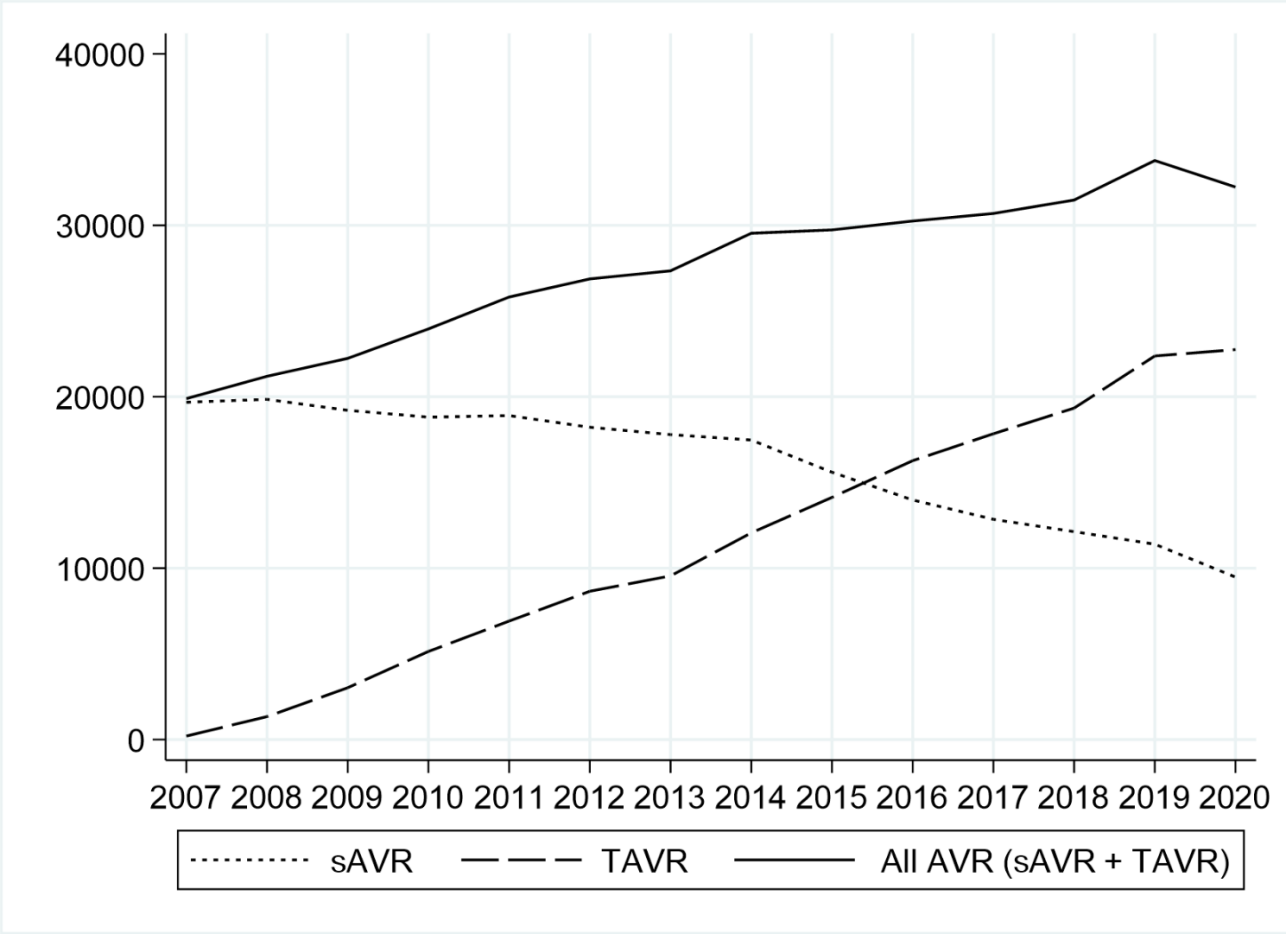
Number of aortic valve replacements in Germany 2007–2020 Number of all aortic valve replacements (AVR) and development of surgical (sAVR) and transcatheter (TAVR) aortic valve replacements in Germany

In Germany, a total of 225,398 isolated sAVR and 159,638 isolated TAVR procedures were conducted between 2007 and 2020 and included in our analysis. As shown in Fig. 1, the yearly number of sAVR procedures declined over the study period, while the number of TAVR procedures increased.

**Fig. 2:**
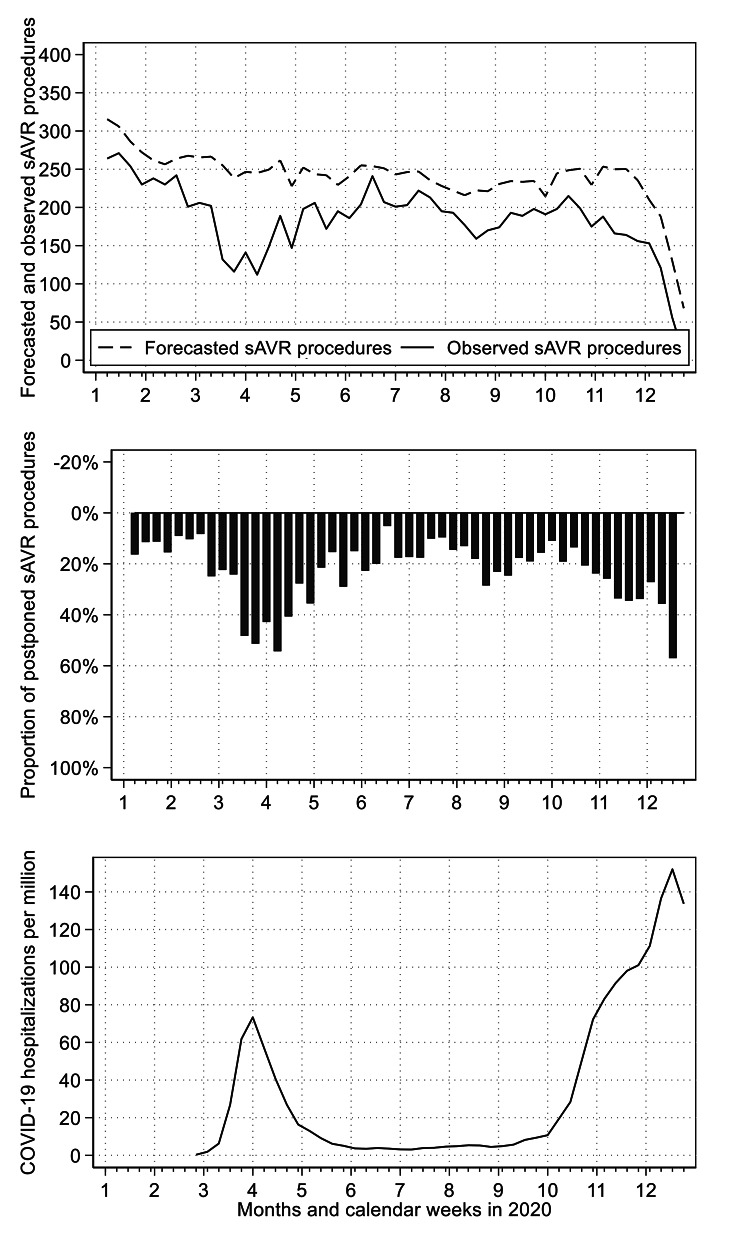
Forecasted and observed surgical aortic valve replacements in 2020 Forecasted and observed weekly number of sAVR procedures (above figure), relative difference between forecasted and observed weekly number of sAVR procedures (middle figure), and weekly number of new hospital admissions for COVID-19 per million inhabitants according to Roser et al. [18] (bottom figure)

Figure 2 compares the forecasted number of sAVR procedures with the observed number of sAVR procedures. For the entire year 2020, the number of sAVR procedures was reduced by 24.63% (95%CI: 20.28–28.99%). During the first wave of the pandemic (week 12–21), the mean weekly reduction was 36.65% (27.30 to 46.00). During the second wave of the pandemic (week 41–52), the mean weekly reduction was 34.65% (21.33–47.99%).

**Fig. 3:**
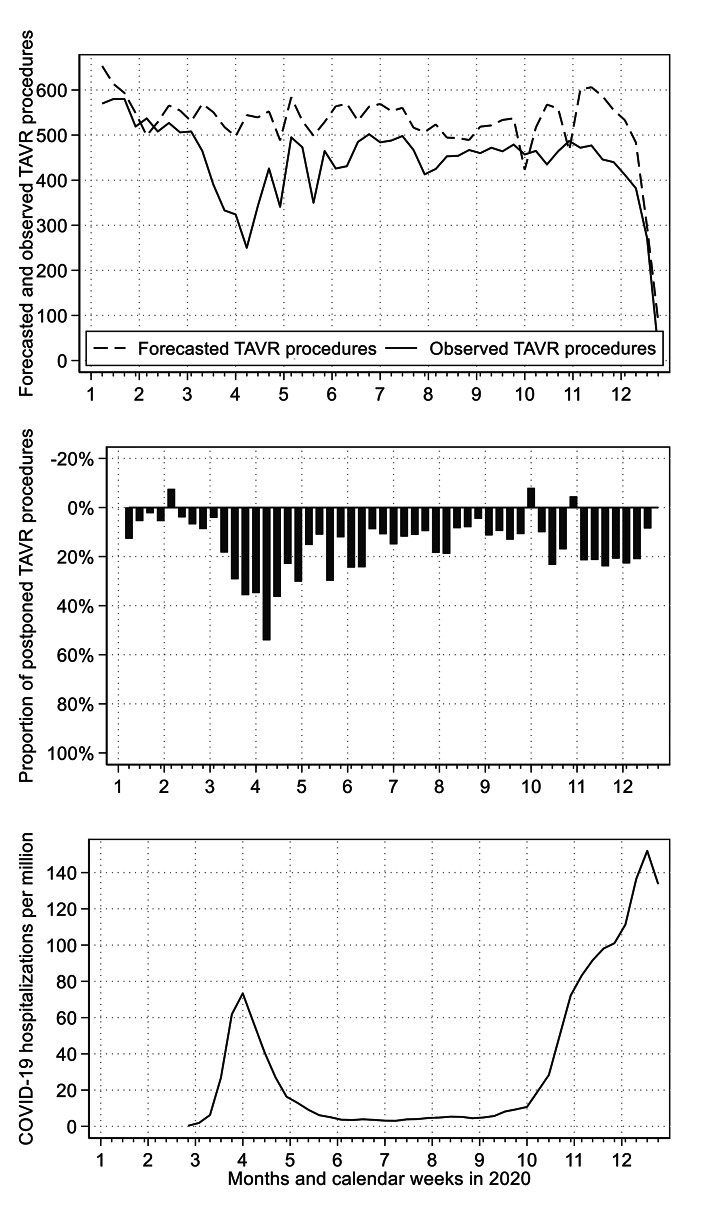
Forecasted and observed transcatheter aortic valve replacements in 2020 Forecasted and observed weekly number of TAVR procedures (above figure), relative difference between forecasted and observed weekly number of TAVR procedures (middle figure), and weekly number of new hospital admissions for COVID-19 per million inhabitants according to Roser et al. [18] (bottom figure)

Figure 3 compares the forecasted number of TAVR procedures with the observed number of TAVR procedures. For the entire year 2020, the number of TAVR procedures were reduced by 16.42% (12.54–20.31%). During the first wave of the pandemic (week 12–21), the mean weekly reduction was 29.94% (21.32–38.55%). During the second wave of the pandemic (week 41–52), the mean weekly reduction was substantially smaller: 21.25% (10.23–32.26%).

**Fig. 4:**
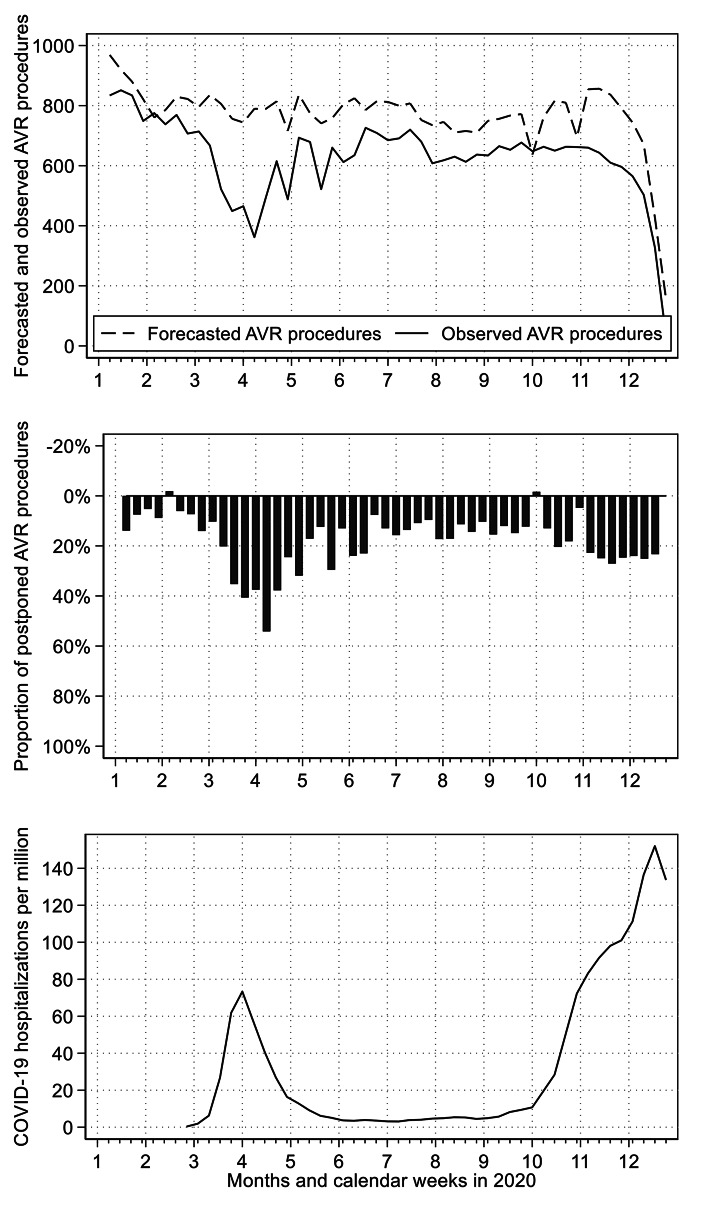
Forecasted and observed surgical and transcatheter aortic valve replacements combined in 2020 Forecasted and observed weekly number of AVR procedures (above figure), relative difference between forecasted and observed weekly number of AVR procedures (middle figure), and weekly number of new hospital admissions for COVID-19 per million inhabitants according to Roser et al. [18] (bottom figure)

Figure 4 sums up the reduction, compared to the forecasted number, in sAVR and TAVR procedures combined. The reduction compared to the forecast in the number of both AVR procedures performed over the entire year 2020 was 19.07% (15.19–22.95%). During the first wave of the pandemic (week 12–21), the mean weekly reduction was 32.06% (23.44–40.68%), and during the second wave of the pandemic (week 41–52), the mean weekly reduction was 25.58% (14.19–36.97%).

## Discussion

During the first wave of COVID-19 infections in March and April in Germany, hospitals were obliged to stop elective surgery to ensure a high capacity in intensive care units [12].

Corresponding to an increasing rate of COVID-19 hospitalizations, the number of AVR treatments was reduced by 32.06% (week 12–21) compared to the forecasted number of procedures. Interestingly, there is no catch-up effect in the following months for sAVR and only a shallow catch-up effect for TAVR. This led to an overall reduction of 19.07% in aortic valve stenosis cases treated in Germany in 2020. Similar findings of a significant decrease in sAVR and TAVR procedures were reported for England and Italy [13, 14].

The hospitalization of COVID-19 patients was increasing rapidly in March 2020 in Germany. Interestingly, the number of surgical aortic valve replacements was already declining in January and February 2020 compared to the forecast. On the other hand, the number of TAVI procedures in these two months was above the level expected from the forecast. This may reflect the trend of increasing numbers of TAVI procedures for aortic valve stenosis in the past years [15].

In November and December 2020, the number of hospitalized COVID-19 patients increased again, leading to a maximum of over 140 COVID-19 patients per million in the last week of 2020. On the other hand, the number of aortic valve treatments decreased less than in the first wave (-25.58% in week 41–51 compared to -32.06% in week 12–21). This may be the result of better preparation by the hospitals and a better focus on interventions that cannot be postponed.

Working out the various hypotheses to explain why AVR procedures were postponed during the COVID-19 pandemic remains challenging. The same is true regarding the potential clinical consequences of this reduced uptake. Basically, there are two possibilities: either patients had fewer hospital visits during COVID-19, or physicians decided to follow up more patients with critical valve disease requiring intervention. In either case, the clinical impact on patients who require intervention but are followed up medically is substantial. As life expectancy increases over the years, it is understandable that there has been an increase in the use of TAVR rather than SAVR in older patients. However, with the COVID-19 pandemic, physicians are putting the option of TAVR versus open-heart surgery on the clinical decision-making agenda. Despite this situation, the decrease in TAVR accrual rates seen during the pandemic is interesting.

There are several limitations to this study. In general, our study design has several distinct limitations compared to retrospective registry studies that have to be taken into account. Traditional registries record more clinical details, especially with regard to diagnoses and procedures that do not affect reimbursement and are therefore frequently not coded by hospital administrators, and with regards to long-term outcome.

However, the analysis of a nationwide administrative dataset has several other advantages and can avoid limitations frequently encountered in traditional registries. As data transfer to Research Data Centres of the Federal Bureau of Statistics is mandatory in Germany, our cohort is virtually complete and avoids selection bias by individual investigators, which tends to result in an underestimation of risk.

Further limitations include: Firstly, we focused on the total number of sAVR and TAVR only in Germany. Secondly, the outcomes of the patients with postponed treatments remain unclear. This point is of critical importance, since the total number of aortic valve procedures declined in 2020. Death due to COVID-19 infection or non-treated aortic valve stenosis is a distinct possibility for these patients, since COVID-19 can be expected to be associated with significant mortality in the predominantly older aortic valve stenosis patient cohort, and untreated symptomatic aortic valve stenosis has a poor prognosis in itself [16, 17].

In conclusion, we showed a significant decrease in the number of sAVR and TAVR procedures performed during the first and second waves of COVID-19 pandemic in Germany. Future investigations will have to determine if there is a link to the outcomes of patients with aortic valve stenosis during the COVID-19 pandemic. Furthermore, this study demonstrated that the share of TAVR among all AVR rose over the past 13 years.

## Conclusion

COVID-19 has caused the deferral of millions of elective procedures. We show that the first year of the COVID-19 pandemic saw a substantial postponing of aortic valve replacement procedures in Germany. Postponing was higher for surgical than for transcatheter aortic valve replacement procedures and less pronounced during the second wave of the COVID-19 pandemic.

## Electronic supplementary material

Below is the link to the electronic supplementary material.

Additional File: **Poisson regression models to predict the number of sAVR and TAVR procedures in 2020**

## Data Availability

The datasets used and/or analysed during the current study available from the corresponding author on reasonable request.

## Abbreviations

sAVR: Surgical aortic valve replacement
TAVR: Transcatheter aortic valve replacement
AVR: Aortic valve replacement
DESTATIS: Research Data Centre of the Federal Bureau of Statistics
ICD-10: ICD, Tenth Revision
