# Supplementary material for: Impact of the COVID-19 pandemic on aortic valve replacement procedures in Germany
Source: BMC Cardiovasc Disord. 2023 Apr 6;23:187. doi: 10.1186/s12872-023-03213-y (PMC10079149; doi:10.1186/s12872-023-03213-y)
Supplement: Supplementary file 1 — Additional File: Poisson regression models to predict the number of sAVR and TAVR procedures in 2020 [file 12872_2023_3213_MOESM1_ESM.pdf]

Supplemental Table 1: Poisson regression models to predict the number of sAVR and TAVR procedures in 2020

sAVR **N=216,398**

Regression 2007-2019

	**Coeff**	**p-value**	**95%CI**	
year	-0.04	0.000	-0.04	-0.04
week				
2	0.33	0.000	0.19	0.47
3	0.30	0.000	0.15	0.44
4	0.23	0.001	0.09	0.37
5	0.18	0.010	0.04	0.32
6	0.14	0.052	0.00	0.28
7	0.12	0.087	-0.02	0.26
8	0.15	0.031	0.01	0.29
9	0.16	0.021	0.03	0.30
10	0.16	0.028	0.02	0.30
11	0.16	0.022	0.02	0.30
12	0.12	0.137	-0.04	0.27
13	0.05	0.532	-0.10	0.20
14	0.08	0.323	-0.08	0.25
15	0.08	0.325	-0.08	0.23
16	0.09	0.231	-0.06	0.25
17	0.14	0.040	0.01	0.28
18	0.00	0.962	-0.16	0.16
19	0.10	0.165	-0.04	0.25
20	0.07	0.373	-0.08	0.22
21	0.06	0.431	-0.10	0.22
22	0.01	0.889	-0.14	0.16
23	0.06	0.411	-0.08	0.20
24	0.12	0.101	-0.02	0.25
25	0.11	0.118	-0.03	0.25
26	0.10	0.158	-0.04	0.24
27	0.07	0.342	-0.07	0.21
28	0.08	0.255	-0.06	0.22
29	0.08	0.238	-0.06	0.22
30	0.04	0.611	-0.11	0.18
31	0.00	0.951	-0.14	0.15
32	-0.02	0.746	-0.16	0.11
33	-0.05	0.474	-0.19	0.09
34	-0.02	0.766	-0.16	0.12
35	-0.03	0.709	-0.16	0.11
36	0.02	0.819	-0.12	0.15
37	0.03	0.663	-0.11	0.18
38	0.03	0.694	-0.11	0.17
39	0.03	0.650	-0.11	0.18
40	-0.06	0.451	-0.20	0.09
41	0.07	0.294	-0.07	0.21
42	0.09	0.193	-0.05	0.23
43	0.10	0.168	-0.04	0.24
44	0.01	0.890	-0.14	0.17
45	0.11	0.121	-0.03	0.25
46	0.10	0.190	-0.05	0.24
47	0.10	0.171	-0.04	0.24
48	0.04	0.600	-0.10	0.18
49	-0.08	0.286	-0.22	0.07
50	-0.19	0.013	-0.34	-0.04
51	-0.55	0.000	-0.75	-0.36
52	-1.20	0.000	-1.54	-0.87
53	-1.65	0.000	-1.97	-1.32
_cons	88.63	0.000	84.29	92.97

TAVR **N=136,890**

Regression 2007-2019

	**Coeff**	**p-value**	**95%CI**	
year	0.15	0.000	0.14	0.15
week				
2	0.45	0.000	0.26	0.64
3	0.39	0.000	0.19	0.58
4	0.35	0.000	0.17	0.54
5	0.28	0.004	0.09	0.47
6	0.18	0.060	-0.01	0.37
7	0.24	0.012	0.05	0.43
8	0.31	0.002	0.11	0.50
9	0.29	0.004	0.09	0.48
10	0.24	0.011	0.05	0.43
11	0.31	0.001	0.12	0.50
12	0.28	0.006	0.08	0.48
13	0.22	0.069	-0.02	0.45
14	0.18	0.115	-0.04	0.40
15	0.27	0.017	0.05	0.49
16	0.26	0.056	-0.01	0.52
17	0.28	0.013	0.06	0.51
18	0.16	0.110	-0.04	0.36
19	0.34	0.001	0.13	0.54
20	0.24	0.014	0.05	0.44
21	0.18	0.061	-0.01	0.37
22	0.24	0.045	0.01	0.47
23	0.30	0.003	0.11	0.50
24	0.31	0.003	0.11	0.52
25	0.24	0.013	0.05	0.44
26	0.30	0.002	0.11	0.50
27	0.31	0.001	0.13	0.50
28	0.28	0.004	0.09	0.48
29	0.30	0.002	0.11	0.48
30	0.22	0.023	0.03	0.40
31	0.20	0.039	0.01	0.38
32	0.23	0.016	0.04	0.42
33	0.17	0.083	-0.02	0.37
34	0.17	0.081	-0.02	0.36
35	0.16	0.109	-0.04	0.36
36	0.22	0.027	0.03	0.41
37	0.23	0.024	0.03	0.42
38	0.25	0.010	0.06	0.44
39	0.25	0.008	0.07	0.44
40	0.02	0.879	-0.19	0.22
41	0.22	0.022	0.03	0.40
42	0.31	0.002	0.11	0.51
43	0.29	0.002	0.11	0.48
44	0.11	0.323	-0.11	0.33
45	0.37	0.000	0.18	0.56
46	0.38	0.000	0.19	0.56
47	0.34	0.000	0.15	0.53
48	0.29	0.003	0.10	0.47
49	0.25	0.010	0.06	0.44
50	0.15	0.122	-0.04	0.34
51	-0.34	0.011	-0.60	-0.08
52	-1.55	0.000	-1.93	-1.17
53	-1.73	0.000	-2.24	-1.23
_cons	-287.27	0.000	-294.79	-279.75

In order to obtain predicted procedure numbers for the year 2020, Stata's margins command was applied

Forecasted sAVR

week	**Coeff**	**p-value**	**95%CI**		**Observed sAVR**
1	227.07	0.000	196.75	257.40	74
2	315.45	0.000	299.45	331.46	264
3	306.05	0.000	287.02	325.09	271
4	286.34	0.000	272.11	300.57	254
5	272.08	0.000	259.97	284.19	230
6	261.62	0.000	246.81	276.44	238
7	256.44	0.000	244.75	268.14	230
8	263.87	0.000	253.78	273.96	242
9	267.61	0.000	255.11	280.11	201
10	265.45	0.000	253.33	277.57	206
11	266.48	0.000	255.63	277.32	202
12	255.20	0.000	234.79	275.61	132
13	238.40	0.000	219.60	257.21	116
14	246.55	0.000	223.15	269.95	141
15	245.18	0.000	225.96	264.41	112
16	249.41	0.000	229.30	269.51	148
17	261.53	0.000	252.81	270.24	189
18	227.96	0.000	207.44	248.48	147
19	252.19	0.000	234.82	269.57	198
20	243.49	0.000	224.23	262.74	206
21	242.15	0.000	219.99	264.31	172
22	229.46	0.000	214.23	244.69	195
23	240.75	0.000	229.29	252.21	186
24	255.04	0.000	243.22	266.86	204
25	254.08	0.000	241.02	267.15	241
26	251.26	0.000	238.47	264.04	207
27	243.13	0.000	230.57	255.68	201
28	246.28	0.000	234.31	258.24	203
29	247.03	0.000	234.97	259.09	222
30	235.65	0.000	221.98	249.32	213
31	228.09	0.000	215.71	240.47	195
32	221.97	0.000	212.82	231.12	193
33	216.00	0.000	207.55	224.45	177
34	222.36	0.000	212.46	232.25	159
35	221.19	0.000	211.41	230.97	170
36	230.71	0.000	222.04	239.37	174
37	234.45	0.000	220.41	248.48	193
38	233.48	0.000	222.91	244.05	189
39	234.69	0.000	221.58	247.81	198
40	214.51	0.000	199.78	229.23	191
41	244.74	0.000	232.83	256.66	198
42	248.65	0.000	239.02	258.28	215
43	250.69	0.000	237.85	263.53	199
44	229.58	0.000	210.63	248.53	175
45	253.49	0.000	241.83	265.15	188
46	249.97	0.000	235.41	264.53	166
47	250.32	0.000	238.49	262.16	164
48	235.62	0.000	225.38	245.85	156
49	210.02	0.000	197.94	222.11	153
50	188.21	0.000	175.69	200.74	121
51	130.39	0.000	112.29	148.48	56
52	68.23	0.000	47.26	89.21	6
53	43.76	0.000	30.83	56.69	

Forecasted TAVR

week	**Coeff**	**p-value**	**95%CI**		**Observed TAVR**
1	416.36	0.000	339.76	492.95	158
2	653.01	0.000	625.12	680.90	570
3	613.65	0.000	568.64	658.67	580
4	593.56	0.000	569.91	617.21	580
5	549.20	0.000	521.75	576.65	519
6	499.01	0.000	474.25	523.77	537
7	528.97	0.000	506.69	551.24	508
8	565.64	0.000	525.56	605.71	527
9	554.50	0.000	514.13	594.86	506
10	530.52	0.000	506.82	554.23	508
11	569.19	0.000	539.91	598.46	465
12	550.74	0.000	508.95	592.53	390
13	517.73	0.000	441.16	594.30	333
14	497.28	0.000	435.47	559.08	324
15	544.39	0.000	477.53	611.25	250
16	539.17	0.000	434.85	643.49	343
17	552.82	0.000	480.03	625.62	426
18	488.40	0.000	453.56	523.24	341
19	584.01	0.000	531.29	636.74	495
20	531.45	0.000	494.92	567.99	473
21	498.70	0.000	474.58	522.83	350
22	529.14	0.000	451.01	607.27	465
23	563.82	0.000	522.53	605.12	426
24	569.52	0.000	517.27	621.77	431
25	531.73	0.000	498.04	565.41	485
26	563.13	0.000	527.02	599.25	502
27	569.13	0.000	551.95	586.31	484
28	553.34	0.000	521.58	585.11	488
29	560.44	0.000	539.70	581.17	498
30	516.42	0.000	500.73	532.12	467
31	506.61	0.000	487.58	525.65	413
32	523.73	0.000	504.44	543.02	425
33	494.45	0.000	461.61	527.29	453
34	493.31	0.000	466.86	519.77	454
35	489.14	0.000	452.56	525.72	467
36	518.79	0.000	483.48	554.10	460
37	521.55	0.000	484.49	558.61	472
38	533.65	0.000	506.17	561.13	464
39	537.08	0.000	513.44	560.72	479
40	423.14	0.000	380.82	465.47	457
41	516.80	0.000	502.57	531.03	465
42	567.70	0.000	527.59	607.82	435
43	559.01	0.000	535.41	582.61	464
44	465.82	0.000	406.74	524.91	487
45	600.89	0.000	569.65	632.13	472
46	606.23	0.000	580.31	632.15	477
47	586.29	0.000	556.33	616.24	446
48	555.33	0.000	533.26	577.40	440
49	533.42	0.000	510.43	556.40	412
50	483.47	0.000	458.58	508.36	382
51	297.27	0.000	242.50	352.04	272
52	88.20	0.000	58.87	117.52	27
53	73.57	0.000	38.95	108.19	
